# Application of Multilabel Learning Using the Relevant Feature for Each Label in Chronic Gastritis Syndrome Diagnosis

**DOI:** 10.1155/2012/135387

**Published:** 2012-06-03

**Authors:** Guo-Ping Liu, Jian-Jun Yan, Yi-Qin Wang, Jing-Jing Fu, Zhao-Xia Xu, Rui Guo, Peng Qian

**Affiliations:** ^1^Laboratory of Information Access and Synthesis of TCM Four Diagnosis, Basic Medical College, Shanghai University of Traditional Chinese Medicine, Shanghai 201203, China; ^2^Center for Mechatronics Engineering, East China University of Science and Technology, Shanghai 200237, China

## Abstract

*Background*. In Traditional Chinese Medicine (TCM), most of the algorithms are used to solve problems of syndrome diagnosis that only focus on one syndrome, that is, single label learning. However, in clinical practice, patients may simultaneously have more than one syndrome, which has its own symptoms (signs). *Methods*. We employed a multilabel learning using the relevant feature for each label (REAL) algorithm to construct a syndrome diagnostic model for chronic gastritis (CG) in TCM. REAL combines feature selection methods to select the significant symptoms (signs) of CG. The method was tested on 919 patients using the standard scale. *Results*. The highest prediction accuracy was achieved when 20 features were selected. The features selected with the information gain were more consistent with the TCM theory. The lowest average accuracy was 54% using multi-label neural networks (BP-MLL), whereas the highest was 82% using REAL for constructing the diagnostic model. For coverage, hamming loss, and ranking loss, the values obtained using the REAL algorithm were the lowest at 0.160, 0.142, and 0.177, respectively. *Conclusion*. REAL extracts the relevant symptoms (signs) for each syndrome and improves its recognition accuracy. Moreover, the studies will provide a reference for constructing syndrome diagnostic models and guide clinical practice.

## 1. Introduction

Although Traditional Chinese Medicine (TCM) and Western Medicine diagnose cases in clinical applications, their theoretical systems are totally different. The hard targets such as laboratory and imaging tests are very important for diagnosing diseases in Western Medicine whereas soft targets are much more important in the clinical diagnosis of TCM. 

The so-called soft targets [1] mainly refer to targets that cannot be accurately measured and with poor repeatability. These targets are subjective and are collected through clinical observation of a doctor or the patient's self-report, which cannot be accurately measured using instruments or directly through other means.

Therefore, collecting information in the diagnosis of TCM is difficult because it cannot be measured accurately, with poor measurement repeatability, and are easily influenced by the study sample and environmental factors.

Given that the soft targets of TCM are subjective, fuzzy, and multidimensional, TCM has been considered as a mystical experience in the scientific world and has not been identified in a wide range.

In recent years, the standardization and objectification of TCM diagnosis have gradually become a research hotspot with the development of mathematical statistics, data mining, and pattern recognition technology.

The studies are revealing the rules between syndromes and the information of four diagnosis: inspection, auscultation and olfaction, inquiring, and palpation, and seeking information of four diagnosis for differential diagnosis or extracting classification rules for syndrome differentiation. The research can provide a reference for clinical syndrome differentiation and reduce the subjectivity and ambiguity of diagnosis.

Some researchers have applied the structural equation model in studying chronic atrophic gastritis. The results show that chronic atrophic gastritis resulting from the most common syndromes correspond with diagnostic targets, which is agreement with the clinical practice of TCM [2]. An improved conjugate gradient learning algorithm is used to create the BP model with three layers for diabetes and nephropathy. The results show its advantages in predicting diabetes and nephropathy [3]. An entropy-based partition method for complex systems is applied to establish endothelial dysfunction diagnostic criteria for Yin deficiency syndrome. Moreover, the experimental results are highly consistent with the findings of clinical diagnosis [4]. Multilabel learning [5] combined with the frequency method is presented to select the symptoms that greatly contribute to coronary heart disease. The results show the improvement in the diagnosis of coronary heart disease. Su et al. [6] employed the correlation coefficient, similarity D, the angle cosine, and spectral similarity to study the correlation between the symptoms (signs) and the five syndromes of liver cirrhosis. The research can provide a basis for differentiating patients with nonspecific clinical manifestations.

Our research group focuses on the standardization and objectification of syndrome in TCM. We applied latent structure models [7] to study the association between symptoms of the spleen system. According to a social network theory, we used the associated density method [8, 9] to analyze the correlation between syndrome-syndrome of coronary heart disease and symptom-syndrome of chronic gastritis.

In the studies on syndrome standardization and objectification mentioned above, most of the algorithms were used to solve problems in diagnosing patients with disjoint syndromes, which belong to single-label learning. However, in clinical practice, strong relevance may be observed among different syndromes. Traditional single-label data-mining techniques, which could only build one model at a time, ignore the fact that one patient may be associated with more than one syndrome. In this study, a novel multilabel learning (MLL) technique is explored to solve this problem. Our group [5] applied a multilabel learning algorithm (ML-kNN) to construct a syndrome model for diagnosing CHD in TCM. The ML-kNN produces better results than the ranking Support Vector Machine (Rank-SVM), BPMLL, and kNN based on three criteria, namely, average precision, coverage, and ranking loss.

Compared with traditional learning methods, multilabel learning more effectively identifies syndrome information in TCM and solves problems such as single samples with several syndromes. However, the relationship between features and class labels is not concerned in multilabel learning.

Chronic gastritis (CG) is a common disease and is classified under spleen and stomach diseases in TCM. According to preliminary studies, we applied the feature selection methods to select significant symptoms (signs) associated with each syndrome in CG. In addition, we further applied multilabel learning algorithm to construct the syndrome models of inquiry diagnosis for CG in TCM to provide a reference for the syndrome standardization and objectification of CG.

In this paper, the first section includes the introduction of the research progress in the field of TCM diagnosis, the purpose of the study, and its significance. In Section 2, we introduce the data-collecting methods, which include a variety of feature selection methods and a multilabel learning method designated as REAL. Standardizing a scale of inquiry information is discussed in the results section. The optimal symptom set is obtained for each syndrome using feature selection. The results of diagnostic models constructed are discussed based on the REAL method. Then the results of REAL are compared with other multilabel learning algorithms.  Section 3, the results of feature selection and diagnostic models are analyzed based on TCM theory. The last section concludes and indicates several issues for future studies.

## 2. Material and Methods

### 2.1. Research Subjects

Chronic gastritis (CG) samples were collected from a clinic, in-patient department, and gastroscopy room of the digestive system department of the Longhua Hospital and the Shuguang Hospital of Shanghai University of Traditional Chinese Medicine, the Xinhua Hospital, the Putuo District Central Hospital, and the Shanghai Hospital of Traditional Chinese Medicine. This work was approved by the Shanghai Society of Medical Ethics. All patients signed an informed consent form. A total of 919 valid subjects were enrolled after excluding cases with TCM inquiry diagnosis scales that lacked information or cannot be diagnosed with CG. Among the 919 patients, 354 were male (38.5%, with an average age of 44.61 yr ± 14.54 yr) and 565 were female (61.5%, with an average age of 48.70 yr ± 12.74 yr).

#### 2.1.1. Inclusion Criteria

Including criteria were

1. patients who meet the diagnostic standards for CG and TCM syndromes, and
2. patients who were informed and have agreed to join this investigation.

#### 2.1.2. Diagnostic Standards

**Western Diagnostic Standards**

The Consensus of National Seminar on CG held by the Chinese Medical Association Digestive Diseases Branch in 2006 [10] was refered to diagnose whether a patient has CG based on gastroscopy results, pathologic results, and clinical performance.

**Chinese Diagnosis Standard**

Diagnosis Standard includes the following eight syndromes (patterns) referring to “Guideline for Clinical Research of New Traditional Chinese Medicine” [11] issued by the Ministry of Health and “National Standard of People's Republic of China: Syndrome Part of TCM Clinical diagnosis and Treatment Terminology” [12] issued by the China State Bureau of Technical Supervision,

1. damp heat accumulating in the spleen-stomach,
2. dampness obstructing the spleen-stomach,
3. spleen-stomach qi deficiency,
4. spleen-stomach cold deficiency,
5. liver stagnation,
6. stagnated heat in the liver-stomach,
7. stomach yin deficiency,
8. blood stasis in the stomach collateral.

#### 2.1.3. Exclusion Criteria

Excluding criteria were

1. mentally ill patients and patients with other severe systemic diseases,
2. patients who have difficulty in describing their conditions, and
3. patients who are not informed or refuse to cooperate.

### 2.2. Method for Establishing TCM Inquiry Diagnosis Scales

The research group was composed of Shanghai senior clinical experts on the digestive system, clinical doctors, and researchers. The final TCM inquiry diagnosis scales were drafted based on past experience in the production of scales [13], a wide range of literature about TCM spleen and stomach diseases, related documents in core magazines, and journals for over 15 years and reports about the frequency of symptoms associated with syndromes in CG diseases in TCM. The scales were also amended and fixed by two rounds of expert consultation and statistical tests. The scales include eight dimensions such as cold or heat, sweat, head, chest and abdomen, urine and stool, diet and taste, sleep, mood, woman aspects and contents of disease history, inspection, and palpation. More than 113 variables were ultimately included in these scales. 

### 2.3. Investigation Methods

The clear definitions of symptoms, the specific methods, and the order of inquiry diagnosis are given in the scales. All samplers must have undergone unified training. The group members assemble regularly and discuss the information of typical patients to ensure the consistency of the collected data.

### 2.4. Diagnosis Methods

Three senior chief doctors with plenty of experience in clinical practices were invited for inquiry diagnosis of the cases in terms of the CG diagnostic standards made by our research group. If two of them have the same diagnosis results, the case was included. Otherwise, the case was not adopted until at least two of them came to the same conclusion.

### 2.5. Data Input and Process Methods

We have the following methods

1. Build a database with Epidata software.
2. Input data two times independently.
3. The Epidata software compares the two data sets and checks out mistakes.
4. Check the investigation form logically in case of filling errors.

### 2.6. Feature Extraction Methods

To obtain the proper set of symptoms for each syndrome, we employed four feature selection methods, namely, mutual information (MI) [14], information gain (IG) [15, 16], conditional mutual information method (CMIM) [17], and minimum redundancy maximum relevance (MRMR) [18], to investigate the relationship between the symptoms and the six common syndromes (patterns), such as the accumulation of damp heat in the spleen-stomach, dampness obstructing the spleen-stomach, spleen-stomach qi deficiency, spleen-stomach cold deficiency, liver stagnation, and stagnated heat in the liver-stomach.

### 2.7. Multilabel Learning Methods

Many real-world problems involving ambiguous objects lose useful information when analyzed using the traditional single-label algorithm. Thus, it will be harmful to the learning performance. To minimize this information loss, multilabel learning was proposed.

Most traditional multilabel classification approaches to learning methods in vector spaces are used based on the assumption that the instances should have the same set of features in the input space for each label. However, for specific labels, not all the features have strong correlations. ML-kNN is the lazy multilabel learning algorithm based on k-nearest neighbor techniques (kNN) [19]. Similar to the kNN algorithm, it finds the k nearest neighbors for each test instance; however, in ML-kNN, the label of each test instance is estimated directly using the k nearest neighbors in instance. We applied a new algorithm called REAL to fit the characteristics for inquiry diagnosis in TCM based on ML-kNN. The REAL algorithm extracts the best feature subset correlated with a certain label as its input space and then calculates the posterior probability combined with the ML-kNN algorithm. The REAL algorithm is shown in Algorithm 1.

### 2.8. Experimental Design and Evaluation

Different characteristics were selected using the REAL algorithm. We selected 112, 100, 70, 60, 50, 40, 30, 20, 10, and 5 symptoms (signs), which correlated with each syndrome to build a syndrome model to study the influence of the different symptoms (signs) on the diagnostic model.

Considering each example could simultaneously be associated with multiple labels, performance evaluation in multilabel learning is different from single-label learning. The following five multilabel evaluation parameters presented in [20] are used in this paper.

**Average Precision**

It evaluates the average fraction of labels ranked above a particular label *y* ∈ *Y*, which actually are in *Y*. The performance is perfect when avgprec_*S*_(*f*) = 1; the bigger the value of avgprec_*S*_(*f*), the better the performance one has.

(1) $$\;\begin{array}{cc} & \text{avgpre}{\text{c}}_{S}\left(f\right)\\ & \;=\frac{1}{p}\overset{p}{\underset{i=1}{\sum }}\frac{1}{\left|Y_{i}\right|}\\ & \;\;\times \underset{y\in Y_{i}}{\sum }\frac{\left|\left\{y^{\prime }\;|\;{\mathrm{rank}}_{f}\left(x_{i},y^{\prime }\right)\leq {\mathrm{rank}}_{f}\left(x_{i},y\right),y^{\prime }\in Y_{i}\right\}\right|}{{\mathrm{rank}}_{f}\left(x_{i},y\right)}. \end{array}$$

**Coverage**

It evaluates how far on the average we need to go down the list of labels to cover all the proper labels of the instance. It is loosely related to precision at the level of perfect recall. The smaller the value of coverage_*S*_(*f*), the better the performance one has.

(2) $$\;\begin{array}{cc} {\text{coverage}}_{S}\left(f\right) & =\frac{1}{p}\overset{p}{\underset{i=1}{\sum }}\underset{y\in Y_{i}}{\max}{\;\mathrm{rank}}_{f}\left(x_{i},y\right)-1,\\ & \;{\mathrm{rank}}_{f}\left(x_{i},y\right)=1-f\left(x_{i},y\right). \end{array}$$

**Ranking Loss**

It evaluates the average fraction of label pairs that are reversely ordered for the instance. The performance is perfect when rloss_*S*_(*f*) = 0; the smaller the value of rloss_*S*_(*f*), the better the performance,

(3) $$\begin{array}{cc} & {\text{rloss}}_{S}\left(f\right)\\ & \;=\frac{1}{p}\overset{p}{\underset{i=1}{\sum }}\frac{1}{\left|Y_{i}\right|\left|\bar{Y_{i}}\right|}\\ & \;\;\times \left|\left\{\left(y_{1},y_{2}\right)\;|\;f\left(x_{i},y_{1}\right)\leq f\left(x_{i},y_{2}\right),\left(y_{1},y_{2}\right)\in Y_{i}\times \bar{Y_{i}}\right\}\right|, \end{array}\;$$

where  $\bar{Y}$ denotes the complementary set of *Y* in *y* · *y* = {1,2,…, *Q*} be the finite set of labels.

**Hamming Loss**

It evaluates how many times instance-label pairs are misclassified; that is, a label not belonging to the instance is predicted, or a label belonging to the instance is not predicted one has,

(4) $$\;{\text{hloss}}_{\Gamma }\left(f\right)=\frac{1}{m}\overset{m}{\underset{i=1}{\sum }}\frac{1}{n}\left|f\left(x_{i}\right)\Delta Y_{i}\right|,$$

where Δ denotes the symmetric difference between two sets.

**One-Error**

It evaluates how many times the top-ranked label is not in the set of proper labels of the instance. The performance is perfect when one-error_Γ_(*f*) = 0 we have.

(5) $${\text{one-error}}_{\Gamma }\left(f\right)=\frac{1}{m}\overset{m}{\underset{i=1}{\sum }}⟦\underset{y\in Y}{\arg\max}f\left(x_{i},y\right)⟧\notin Y_{i}.$$

For any predicted *π*, ⟦*π*⟧ equals 1 if *π* holds and 0 if otherwise. Note that, for single-label classification problems, a one-error is identical to an ordinary classification error.

## 3. Results and Discussion

### 3.1. The Results of the Finest Symptoms (Signs) Subsets

#### 3.1.1. The Results of Finest Subsets of Specific Symptoms (Signs)

In the research of multilabel classification of syndrome diagnosis for CG, feature selection methods such as mutual information, IG, CMIM, and MRMR were combined with multilabel learning. The prediction accuracy was highest when 20 features were selected for classification. Based on the results, the features selected by IG are more suitable for TCM theory than those using other algorithms.

- 12 specific symptoms (signs), including yellow tongue coating and greasy tongue coating, were extracted for the pattern of damp heat accumulating in the spleen-stomach.
- 12 specific symptoms (signs), including white and greasy tongue coating, were extracted for the pattern of dampness obstructing the spleen-stomach.
- 11 specific symptoms (signs), including fatigue and tongue with teeth marks, were extracted for the pattern of spleen-stomach qi deficiency.
- 8 specific symptoms (signs), including cold limbs and preference for warm temperature, were extracted for the pattern of spleen-stomach cold deficiency.
- 9 specific symptoms (signs), including white aggravating after anxiety or anger, distending pain in the chest, and hypochondriac area, were extracted for the pattern of liver stagnation.
- 12 specific symptoms (signs), including burning pain and red tongue, were extracted for the pattern of stagnated heat in liver-stomach.

The detailed information about symptoms (signs) is displayed in Table 1.

#### 3.1.2. The Results of Finest Subsets of Negative Symptoms (Signs)

They are as follows

- 8 negative symptoms (signs) including white and thin tongue coating were extracted for the pattern of damp-heat accumulating in the spleen-stomach.
- 12 negative symptoms (signs) including red and dark red tongue were extracted for the pattern of dampness obstructing the spleen-stomach.
- 11 negative symptoms (signs) including thick tongue coating, mixed yellow, and white tongue coating were extracted for the pattern of spleen-stomach qi deficiency.
- 8 negative symptoms (signs) including red lips and good appetite, but easily gets hungry, were extracted for the pattern of spleen-stomach cold deficiency.
- 9 negative symptoms (signs), including fatigue and pain when exposed to cold, were extracted for the pattern of liver stagnation.
- 12 negative symptoms (signs) including tongue with teeth marks and fat tongue were extracted for the pattern of stagnated heat in liver-stomach.

The detailed information about negative symptoms (signs) is displayed in Table 2.

### 3.2. The Results of Syndrome Classification Using Multilabel Learning Methods

#### 3.2.1. Comparison of Average Accuracy with Different Number of Features

Using the REAL algorithm, we selected 112, 100, 70, 60, 50, 40, 30, 20, 10, and 5 symptoms (signs), which correlated with each syndrome to build a syndrome classification model to study the influence of the different symptoms (signs) on the diagnostic model.

**Figure 1:**
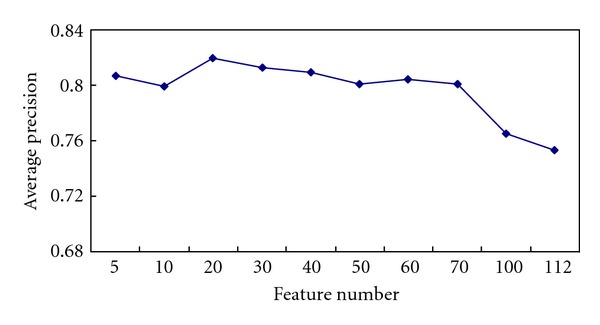
The average accuracy rate with different number of symptoms (signs) by using REAL methods.

The abscissa represents the number of the selected features, and the vertical axis represents their prediction accuracy in Figure 1.

As shown in Figure 1, the average accuracy changes with the number of symptoms (signs). When the number of selected symptoms (signs) was 20, the average accuracy peaked at 82%. Then, it decreased gradually with increasing number of symptoms (signs).

#### 3.2.2. Comparison of Performance of Different Multilabel Learning Algorithms

We selected 20 symptoms (signs) to build the models and compared the five evaluation parameters obtained using ML-kNN, Ensembles of Classifier Chains (ECCs), BSVM, BP-MLL, Rank-SVM, and REAL algorithms. The result is shown in Table 3.

As indicated in Table 3, the highest was 82%, obtained by REAL, whereas the lowest average precision was 54%, obtained using BP-MLL. For the indicators coverage, hamming loss, and ranking loss, the values obtained using the REAL algorithm were lowest at 0.160, 0.142, and 0.177, respectively. In summary, the results obtained using the REAL algorithm were the most accurate.

#### 3.2.3. The Comparison of Accuracy Rates of Various Syndromes Using Different Multilabel Methods (the 20 Features Are Selected in REAL Method)

The results of the REAL method were compared with the other multilabel learning methods, namely, BP-MLL, Rank-SVM, ECC, BSVM, and ML-kNN. The recognition accuracies of the six common syndromes of CG are shown in Table 4.

As shown in Table 4, for the pattern of damp heat accumulation in the spleen-stomach, the REAL algorithm achieved the highest accuracy rate, followed by ECC, BSVM, Rank-SVM, ML-kNN, and BP-MLL. For the pattern of dampness obstructing the spleen-stomach, the REAL algorithm also had the highest accuracy rate, followed by BSVM, ECC, Rank-SVM, ML-kNN, and BP-MLL.

For the pattern of spleen-stomach qi deficiency, the accuracy rate obtained from ECC was the highest, followed by BSVM, REAL, ML-kNN, Rank-SVM, and BP-MLL. For the pattern of spleen-stomach cold deficiency, REAL, ML-kNN and BP-MLL had the highest accuracy rate at 96.6%, followed by ECC, BSVM, and Rank-SVM. For the pattern of liver stagnation, the REAL algorithm achieved the highest accuracy rate, followed by BP-MLL, ML-kNN, BSVM, ECC, and Rank-SVM. For the pattern of stagnated heat in the liver-stomach, BP-MLL and REAL algorithm achieved the highest accuracy rate, followed by ML-kNN, ECC, BSVM, and Rank-SVM.

From the results, the comprehensive performance of REAL method was the best, with the accuracy rates in the six syndromes, except for the pattern of spleen-stomach qi deficiency.

### 3.3. Discussion

A syndrome is a unique TCM concept. It is an abstractive conception of a variety of symptoms and signs. It is a pathological summarization of a certain stage of a disease, and it covers disease location, etiology, and the struggle between the body's resistance and pathogenic factors. Different syndromes have different clinical manifestations.

Symptoms, which are the external manifestations of a disease and a syndrome, refer to subjective abnormalities and the abnormal signs of patients elicited by doctors using the four diagnostic methods.

The etiology, location, nature, the struggle between the body's resistance and pathogenic factors, and the condition at a certain stage of the disease process are highly summarized using syndrome differentiation. Syndrome differentiation involves three steps: (a) determining symptoms and signs through inspection, auscultation, inquiry, and palpation; (b) making an overall analysis of the information; (c) making a diagnostic conclusion. All these steps are based on TCM theory.

**Figure 2:**
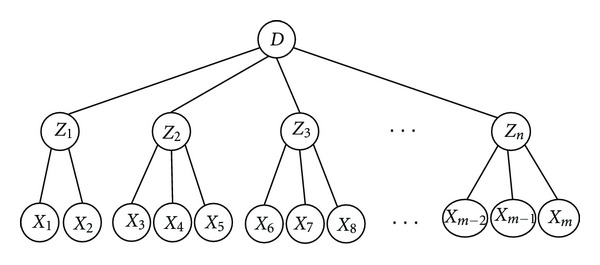
Syndrome diagnostic schemes.

Figure 2 shows the TCM diagnosis of the network structure diagram. Network structure can be compared to a tree, where the root node is composed of a number of leaf nodes. *X*1, *X*2,…, *X*7 leaf nodes are directly observed, and we call them manifest variables, which denote the symptoms and signs in TCM. *Z*1, *Z*2, *Z*3, and *Z*4 are the root nodes that are indirectly measured through their manifestations, and we call them latent variables, which represent the syndromes of chronic gastritis in TCM. The syndrome can be observed alone or with others, such as *Z*1 and *Z*2, or *Z*2 and *Z*3, which may appear together.

*D* denotes the disease. In this study, it represents chronic gastritis, which is a disease defined in Western Medicine. Chinese medical diagnosis of chronic gastritis may contain syndromes like latent variables *Z*1, *Z*2, *Z*3 … and so on.

#### 3.3.1. The Finest Symptoms (Signs) Feature Subsets for Each Syndrome

Feature selection is a hot topic in the field of machine learning. It studies how to select the most effective feature subset from a set of original feature sets to reduce the feature space dimension and enhance the generalization ability of the model.

Feature selection not only removes the redundant and irrelevant features of the data, but also significantly reduces the cost of data mining.

Information gain is a widely used feature selection method [21]. It was first proposed for text classification and was then used in other areas such as image processing and bioinformatics.

Currently, those feature selection methods [22] have been used in TCM diagnosis for selecting symptoms (signs) and building diagnostic models. Many studies have shown that these feature selection methods select key features effectively and also remove irrelevant features. Some symptoms and signs in TCM have certain specific meanings that can be used for determining the syndrome.

When making a diagnosis is difficult using positive aspects, doctors can diagnose by eliminating symptoms and signs of similar syndromes.

Negative information [23] denotes some symptoms that have a negative meaning in the diagnosis of certain diseases, or some information that are impossible to be observed in some diseases.

The purpose of this study is to recognize the common syndromes of CG using IG combined with multilabel learning. The six finest symptoms (signs) subsets were selected by correlating the six common syndromes of CG, which include the specific and negative symptoms (signs). The experimental results show that the six finest symptoms (signs) subsets are basically in accordance with the TCM theory, clinical practice, and the previous Chinese diagnostic standard.

However, individual symptoms (signs) such as the preference for pressure and warm temperature, fixed pain with the syndrome of liver depression and qi stagnation do not agree with the TCM theory, which may be due to the fact that several syndromes appear together.

#### 3.3.2. Comparison between REAL and Other Multilabel Learning Methods

Compared with conventional learning methods, multilabel learning identifies syndromes in TCM more effectively and solves problems of one sample being associated with several syndromes.

In clinical practice, relevance among different syndromes may exist. The syndrome complex of one patient is mainly composed of several syndromes. For example, spleen-stomach qi deficiency syndrome usually exists with dampness obstructing the middle energizer syndrome, qi stagnation syndrome, turbid phlegm syndrome, or blood stasis syndrome.

In multilabel data, there is a relationship among labels. However, this relationship may be bound to be ignored inevitably by using the single-label learning. For this reason, multilabel learning algorithms are developed to facilitate the correlation of the labels.

Compared with other traditional multilabel learning methods, the REAL algorithm found the relevant symptom subset of each syndrome with feature selection. Moreover, the REAL algorithm identified the syndrome information of CG in TCM more effectively and accurately.

In addition, the REAL algorithm assisted in extracting the corresponding specificity and negative symptoms (signs) through feature selection. Extracted features are not only used for identifying the syndrome of chronic gastritis, but it also improves the syndrome diagnostic accuracy of chronic gastritis.

## 4. Conclusions

To fully understand the characteristics of multilabel data of TCM in syndrome diagnosis, feature selection was combined with a multilabel learning algorithm.

Applying the REAL method extracts the relevant symptoms (signs) for each syndrome and improves the accuracy of syndrome diagnosis in CG.

The study showed that the six finest symptoms (signs) subsets agree with the theory and clinical practice of TCM. In addition, the study will serve as references for establishing diagnostic criteria and a diagnostic model for CG and a better guide for clinical practice. Further studies will focus on building an intelligent diagnostic system for CG with application of the method on biomedical data sets.

## Figures and Tables

**Algorithm 1:**
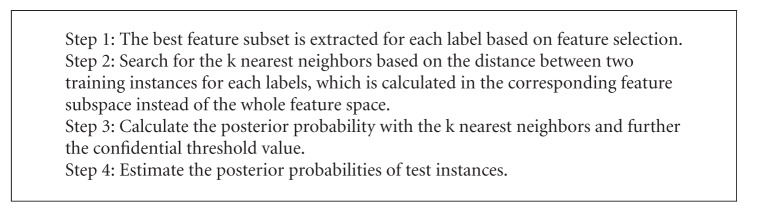
REAL algorithm.

**Table 1:** The finest subsets of specific symptoms (signs).

Symptoms (signs)			Syndromes (patterns)			
	Damp-heat accumulating in the spleen-stomach	Dampness obstructing the spleen-stomach	Spleen-stomach qi deficiency	Spleen-stomach deficiency cold	Liver stagnation	Stagnated heat in liver-stomach
1	Yellow tongue coating	Greasy tongue coating	Fatigue	Cold limbs	Aggravating after anxiety or anger	Red tongue

2	Greasy tongue coating	Thick tongue coating	White tongue coating	Preference for warm	Distending pain in the chest and hypochondriac area	Burning pain

3	Red tongue	White tongue coating	Tongue with teethmarks	White tongue coating	Belching	Distending pain in the chest and hypochondriac area

4	Thick tongue coating	Whitish tongue	Pale-white tongue	Cold pain	Pain of unfixed location	Preference for cold

5	Retrosternal burning sensation	Tongue with teethmarks	Fat tongue	Whitish tongue	Gastric distension	Yellow tongue coating

6	Dry tongue coating	Fat tongue	Whitish lips	Loose stool	Aggravating after diet	An empty sensation in the stomach

7	Greasy taste	Dark-red tongue	Loose stool	Heaviness of the body	Preference for pressure	Dry stool

8	Dark-red tongue	Slippery tongue coating	Dizziness	Thin tongue coating	Preference for warm	Thin tongue

9	Mixed yellow and white tongue coating	Slippery pulse	Thin tongue coating		Fixed pain	Thirsty

10	Bitter taste in the mouth	Cold limbs	Heaviness of the body			Red lips

11	Preference for cold	Bluish or purple tongue	Whitish complexion			Soure taste

12	Slippery pulse	Hesitant pulse				Insomnia

**Table 2:** The finest subsets of negative symptoms (signs).

Symptoms (Signs)			Syndromes (Patterns)			
	Damp-heat accumulating in the spleen-stomach	Dampness obstructing the spleen-stomach	Spleen-stomach qi deficiency	Spleen-stomach deficiency cold	Liver stagnation	Stagnated heat in liver-stomach
1	White tongue coating	Red tongue	Red lips	Red lips	Fatigue	Tongue with teethmarks

2	Thin tongue coating	Dark-red tongue	Thick tongue coating	Stabbing pain	Thick tongue coating	Thick tongue coating

3	Fat tongue	Thin tongue coating	Mixed yellow and white tongue coating	Good appetite but fast hunger	Bitter taste	Greasy tongue coating

4	Tongue with teethmarks	Yellow tongue coating	Greasy tongue coating	Thick tongue coating	Cold pain	Fat tongue

5	Whitish Lips	Distending pain in the chest and hypochondriac area	Red tongue	Fetid mouth odor	Greasy tongue coating	Whitish tongue

6	Whitish complexion	Wiry pulse	Dark-red tongue	Red tongue	Rapid pulse	White tongue coating

7	Whitish tongue	Whitish lips	Yellow tongue coating	Heat sensation in both palms and soles	Thin tongue coating	Slippery pulse

8	Dark -purple lips	Yellow urine	Retrosternal burning sensation	Thin tongue	Loose stool	Cold limbs

9			Large pulse	Yellow tongue coating	Deep pulse	

10				Preference for eating cold food	Heaviness of the body	

11				Dry tongue coating	Rotten tongue coating	

12				Hesitant pulse		

**Table 3:** Performance of different multilabel learning algorithms.

Group (mean ± std)	ML-kNN	ECC	BSVM	BP-MLL	RANK-SVM	REAL
Average precision	0.759 ± 0.029	0.802 ± 0.016	0.802 ± 0.016	0.540 ± 0.023	0.707 ± 0.022	0.820 ± 0.029
Coverage	0.200 ± 0.023	0.186 ± 0.019	0.174 ± 0.023	0.345 ± 0.039	0.237 ± 0.016	0.160 ± 0.020
Hamming loss	0.167 ± 0.014	0.148 ± 0.016	0.156 ± 0.014	0.304 ± 0.014	0.214 ± 0.014	0.142 ± 0.019
One error	0.375 ± 0.050	0.261 ± 0.024	0.307 ± 0.022	0.755 ± 0.029	0.449 ± 0.034	0.283 ± 0.055
Ranking loss	0.167 ± 0.025	0.190 ± 0.025	0.130 ± 0.017	0.334 ± 0.040	0.206 ± 0.014	0.117 ± 0.018

**Table 4:** Comparison of recognition accuracy for six common syndromes.

Syndromes (Patterns)	ML-kNN	ECC	BSVM	BP-MLL	Rank-SVM	REAL
Damp-heat accumulating in the spleen-stomach	0.869 ± 0.036	0.899 ± 0.025	0.884 ± 0.025	0.247 ± 0.035	0.880 ± 0.028	0.901 ± 0.030
Dampness obstructing the spleen-stomach	0.737 ± 0.044	0.789 ± 0.052	0.800 ± 0.035	0.683 ± 0.052	0.762 ± 0.044	0.830 ± 0.038
Spleen-stomach qi deficiency	0.689 ± 0.065	0.741 ± 0.037	0.712 ± 0.023	0.538 ± 0.039	0.679 ± 0.068	0.699 ± 0.041
Spleen-stomach deficiency cold	0.966 ± 0.017	0.958 ± 0.019	0.943 ± 0.027	0.966 ± 0.017	0.793 ± 0.036	0.966 ± 0.023
Liver stagnation	0.827 ± 0.056	0.820 ± 0.043	0.826 ± 0.049	0.831 ± 0.054	0.801 ± 0.047	0.840 ± 0.063
Stagnated heat in liver-stomach	0.908 ± 0.023	0.906 ± 0.034	0.901 ± 0.030	0.910 ± 0.022	0.799 ± 0.048	0.910 ± 0.019
